# NGS-Based Genomic Profiling Identifies Independent Predictors of Time to Castration Resistance in Hormone-Sensitive Prostate Cancer: A Retrospective Real-World Study

**DOI:** 10.3390/curroncol33070416

**Published:** 2026-07-10

**Authors:** Merve Turan, Merve Çırak Balta

**Affiliations:** 1 Department of Medical Oncology, Faculty of Medicine, Adnan Menderes University, Aydın 09010, Türkiye; 2 Department of Pathology, Faculty of Medicine, Adnan Menderes University, Aydın 09010, Türkiye; merve.cirak.balta@adu.edu.tr

**Keywords:** prostate cancer, castration-resistant prostate cancer, next-generation sequencing, *KMT2C*, genomic profiling, hormone-sensitive prostate cancer, mismatch repair deficiency, biomarker

## Abstract

The prognostic significance of next-generation sequencing (NGS) findings during the hormone-sensitive phase of prostate cancer remains incompletely characterized. This retrospective cohort study included 92 patients who underwent NGS analysis on tumor tissue between 2019 and 2025. The primary endpoint was time to castration-resistant prostate cancer (CRPC) from androgen deprivation therapy (ADT) initiation; secondary endpoints were overall survival from ADT initiation (OS-ADT) and from diagnosis. Kaplan-Meier and Cox regression analyses were performed. CRPC developed in 66 patients (71.7%) at a median of 21.1 months. The most frequently altered genes were *ATR* (35.9%), *PTEN* (28.3%), *TP53* (26.1%), and *BRCA2* (15.2%). *KMT2C* alteration (5.4%) was the strongest independent genomic predictor of shorter time to CRPC (HR = 6.804, *p* = 0.003) and OS-ADT (HR = 4.730, *p* = 0.019). *TP53* alteration independently predicted shorter OS-ADT (HR = 1.810, *p* = 0.038). High genomic burden independently predicted shorter time to CRPC (HR = 1.917, *p* = 0.032). Homologous recombination repair deficiency was not associated with outcomes, attributable to high *ATR* alteration frequency introducing pathway heterogeneity. Mismatch repair deficiency showed a borderline association with shorter OS-ADT (20.7 vs. 44.0 months; *p* = 0.060). An exploratory composite risk score stratified patients into three prognostic groups with markedly different outcomes (HR = 7.904, *p* = 0.001). NGS analysis during the hormone-sensitive phase identifies independent predictors of castration resistance, supporting its integration at ADT initiation for risk stratification and biomarker-guided treatment planning.

## Simple Summary

Prostate cancer is one of the most common cancers in men, and most patients eventually develop resistance to standard hormone-blocking treatments—a stage called castration-resistant prostate cancer. Predicting which patients will progress quickly remains a major clinical challenge. In this study, we analyzed tumor DNA from 92 prostate cancer patients using next-generation sequencing, a technology that reads thousands of genes simultaneously to identify genetic changes that could predict faster progression to castration resistance. We found that alterations in the *KMT2C* gene—which normally acts as a molecular brake on cancer cell transformation—were the strongest predictor of early resistance, with affected patients progressing nearly four times faster than those without this alteration. Patients with defects in mismatch repair genes, while not progressing faster, had significantly shorter survival, highlighting their candidacy for immunotherapy. These findings support incorporating genetic tumor profiling at the time of diagnosis to guide personalized treatment decisions.

## 1. Introduction

Globally, prostate cancer (PCa) ranks as the second-most frequently diagnosed malignancy in men, accounting for approximately 1.4 million new cases in 2022 [1]. In parallel with this global burden, the incidence of PCa in Türkiye was documented at 40.3 per 100,000 in 2018 [2]. Androgen deprivation therapy (ADT) remains the standard of care for metastatic hormone-sensitive prostate cancer (mHSPC); however, most patients progress to castration-resistant prostate cancer (CRPC) within 18–24 months [3,4]. This transition—defined by prostate-specific antigen (PSA) and/or radiological progression despite castrate serum testosterone levels (<50 ng/dL)—carries a poor prognosis even in the contemporary treatment era [4,5].

Classical prognostic parameters, including PSA kinetics and Gleason grade, are useful in clinical practice, but they do not reliably predict how quickly resistance will develop in individual patients [6,7]. Next-generation sequencing (NGS) can characterize the molecular changes associated with treatment escape more directly [8,9]. Landmark genomic characterization of metastatic CRPC has demonstrated that aberrations in AR, *TP53*, *PTEN*, and *RB1* are among the most frequently altered genes in advanced disease [10]. Persistent AR signaling through gene amplification, ligand-binding domain mutations, or splice variant expression (notably AR-V7) represents the dominant ADT escape mechanism [10,11]. Compound *TP53* and *RB1* loss drives lineage plasticity via *SOX2* upregulation, enabling phenotypic shift toward AR-independent cell states; concurrent loss was identified in 39% of mCRPC adenocarcinomas and 74% of neuroendocrine-like CRPC cases [12].

Alterations in DNA damage repair (*DDR*) and homologous recombination repair *(HRR)* genes are detected in approximately 19–23% of mCRPC cases [10]. The PROfound phase III trial established that the PARP inhibitor olaparib significantly extended progression-free survival in *BRCA1*/2- and *ATM*-altered mCRPC (median 7.4 vs. 3.6 months; hazard ratio [HR] 0.34; *p* < 0.001), making NGS-guided patient selection a clinical standard [13]. Annala et al. further showed that *BRCA2*/*ATM* mutations and *TP53* defects independently predict primary resistance to first-line abiraterone and enzalutamide [11].

Despite these advances, the prognostic relevance of NGS-detected alterations with respect to time to CRPC—rather than mCRPC outcomes—remains incompletely characterized. Population-level genomic data from Turkish and non-Western cohorts are also virtually absent [14]. Therefore, we retrospectively compared NGS panel results obtained during the hormone-sensitive phase with time to CRPC in a real-world Turkish academic cohort. Our aim was to assess whether genomic alterations detected at this earlier stage could help predict early castration resistance. In contrast to previous studies that mainly examined patients after castration resistance had already developed, our study focuses on the hormone-sensitive phase and uses time to CRPC as the primary endpoint. To our knowledge, this is also the first report of real-world genomic profiling data from a Turkish prostate cancer cohort at this stage [2,14].

## 2. Materials and Methods

### 2.1. Study Design and Setting

This was a single-center, observational, retrospective cohort study conducted at Aydın Adnan Menderes University (ADÜ) Application and Research Hospital Medical Oncology Clinic (January 2019–December 2025), following institutional ethics committee approval.

### 2.2. Eligibility Criteria

Inclusion criteria: (1) histopathologically confirmed prostate adenocarcinoma; (2) initiation of ADT or prior surgical castration; (3) availability of NGS panel analysis on tumor tissue; and (4) minimum 12 months of follow-up from ADT initiation. Patients were excluded if NGS analysis could not be completed due to insufficient tissue, or if the ADT initiation date or CRPC development date was unavailable.

### 2.3. Data Collection

We recorded the following variables: age at diagnosis, initial serum PSA, International Society of Urological Pathology (ISUP) grade group, clinical TNM stage, presence of de novo metastatic disease, initial treatment regimen, date of ADT initiation, date of CRPC development or last follow-up, and NGS panel results. Initial treatment was categorized as ADT alone, ADT plus an androgen receptor pathway inhibitor (ARPI; abiraterone acetate, enzalutamide, apalutamide, or darolutamide), or ADT plus docetaxel. Clinical T and N stage and Eastern Cooperative Oncology Group (ECOG) performance status were not consistently documented, so these variables were excluded from the analysis. Baseline PSA values were missing for 20 patients (21.7%); these patients were excluded from PSA-related analyses.

### 2.4. Tumor Tissue and NGS Panel Analysis

NGS panel results were obtained from the pathology laboratory archives. Tumor material came from formalin-fixed paraffin-embedded (FFPE) tissue blocks. In 86 patients (93.5%), NGS was performed on primary prostate biopsy specimens; in the remaining 6 patients (6.5%), metastatic biopsy specimens were used. After consultation with the molecular pathology unit, 10-micron sections were prepared from the most representative tumor-containing block. DNA was then isolated from these sections and analyzed with the QIAseq Targeted DNA Panel (QIAGEN, Hilden, Germany) on an Illumina MiniSeq platform, using paired-end 151bp reads. The panel included 93 genes and 48,831 primers. Covered genes included *BRCA1*, *BRCA2*, *ATM*, *ATR*, *AR*, *TP53*, *PTEN*, *RB1*, *KMT2C*, *MRE11*, *CHEK2*, *NF1*, *SPOP*, and *PIK3CA*, among others. Unique molecular identifiers (UMIs) were assigned to each original DNA molecule before target enrichment. This step was used to support more accurate variant calling and reduce errors related to PCR amplification. Library preparation followed manufacturer protocols; target enrichment was performed using locus-specific and universal primers. Data analysis was performed using the QIAseq pipeline and variants were interpreted using Qiagen Clinical Insight-Interpret (QCI-Interpret, version 9.5.4.20260422) bioinformatics software. Both tools are publicly accessible via the QIAGEN bioinformatics platform (https://www.qiagen.com (accessed on 25 May 2026); https://www.qiagenbioinformatics.com (accessed on 25 May 2026)). For each patient, the presence or absence of a pathogenic or likely pathogenic alteration in each gene was recorded as a binary variable (mutated vs. wild-type).

#### 2.4.1. Genomic Grouping and Pathway-Based Variables

Individual gene alterations were aggregated into pathway-based binary variables. A patient was considered positive for a pathway group if at least one pathogenic or likely pathogenic alteration was detected in any gene within that pathway.

*HRR* deficiency was defined using a restricted gene set (*BRCA1*, *BRCA2*, *ATM*, *ATR*, *BRIP1*, *PALB2*, *RAD50*, *RAD51*, *NBN*, *MRE11*, *ABRAXAS1*, *FANCD2*, *FANCG*, *BLM*, *ERCC5*), consistent with PROfound trial criteria [13]. A broader *DDR* deficiency variable additionally incorporating *CDK12* and *CHEK2* was constructed for sensitivity analyses [10,11]. Mismatch repair (*MMR*) deficiency was defined by alterations in *MLH3*, *MSH3*, *MSH6*, *PMS1*, *PMS2*, *POLD3*, or *POLE*. Pathway-level variables were also constructed for *AR* signaling (*AR*, *SPOP*, *NCOR1*, *HOXB13*, *MED12*), the *PI3K/AKT/PTEN* axis *(PTEN*, *PIK3CA*, *AKT1*, *NF1)*, *WNT* signaling (*APC*, *CTNNB1*, *AXIN2*, *TCF7L2*), *chromatin modifiers (KMT2C*, *KMT2D*, *ARID1A*, *CHD1*, *CREBBP*, *BAP1*, *PBRM1*), and *TGF-β* signaling (*TGFB1*, *TGFBR2*, *SMAD4*). *TP53* and *RB1* alterations were analyzed individually and as a concurrent loss composite, per Mu et al. [12]. Genes with insufficient alteration frequency (*n* < 5) were excluded from inferential analyses.

#### 2.4.2. Composite and Derived Variables

Several composite and derived variables were constructed posthoc in an exploratory manner based on primary survival analyses.

Metastatic status-integrated genomic variables were created for *KMT2C* and *TP53* mutations separately, classifying patients into three groups: (1) wild-type and non-metastatic, (2) wild-type and de novo metastatic, and (3) mutation-positive (regardless of metastatic status).

A multi-pathway genomic burden score was calculated by summing the number of dysregulated pathways per patient across seven pathway-level binary variables (*HRR*, *MMR*, *AR* signaling, *PI3K/AKT*/*PTEN*, *WNT*, *chromatin modifier*, and *TP53*). Based on the distribution of scores in the cohort, patients were grouped as having low genomic burden if 0–1 pathways were affected, and high genomic burden if 2 or more pathways were affected. Although *TP53* was also analyzed individually, it was included in the burden score as a representative of the p53 tumor suppressor pathway.

We defined the high-risk genomic group as patients with at least one alteration in *KMT2C*, *TP53*, or *MRE11*. These genes were selected because each showed individual statistical significance in the primary time-to-CRPC analyses.

An exploratory composite clinical-genomic risk score was constructed by summing five binary variables: *KMT2C* mutation, *TP53* mutation, de novo metastatic disease, high ISUP grade (grade group 4–5), and high genomic burden (≥2 pathways), yielding a score of 0–5. Patients were categorized as low risk (score 0–1), intermediate risk (score 2), or high risk (score ≥ 3).

### 2.5. Primary and Secondary Endpoints

The primary endpoint was time from the start of ADT to the development of CRPC. CRPC was defined per Prostate Cancer Working Group 3 (PCWG3) criteria: three or more consecutive PSA rises (each ≥25% above nadir and ≥2 ng/mL) at intervals of ≥1 week, and/or radiological progression (≥2 new bone lesions on scintigraphy or soft-tissue progression per RECIST 1.1), with castrate testosterone levels (<50 ng/dL) [15]. Patients who had not developed CRPC were censored at the date of their last follow-up. Secondary endpoints included overall survival (OS) calculated from the start of ADT (OS-ADT), OS calculated from the date of diagnosis (OS-diagnosis), and exploratory analyses testing composite clinical-genomic variables as predictors of time to CRPC and OS.

### 2.6. Statistical Analysis

All analyses were performed using SPSS version 26.0 (IBM Corp., Armonk, NY, USA). Continuous variables are reported as mean ± standard deviation (SD) or median (interquartile range [IQR]) based on Shapiro-Wilk normality testing; categorical variables as frequencies and percentages. Group comparisons used the independent samples t-test or Mann-Whitney U test for continuous variables, and chi-square or Fisher’s exact test for categorical variables. Survival curves were estimated by the Kaplan-Meier method and compared with the log-rank test. Univariable and multivariable Cox proportional hazards regression analyses were performed; variables with *p* < 0.10 in univariable analyses were eligible for multivariable models. Multiple models were constructed to address collinearity; the best-fitting model was identified by the lowest −2 log-likelihood (−2LL) value. Results are expressed as HRs with 95% confidence intervals (CIs). Statistical significance was set at *p* < 0.05 (two-tailed).

### 2.7. Ethical Considerations

The study was conducted in accordance with the Declaration of Helsinki, and approved by the Non-Interventional Clinical Research Ethics Committee of Aydın Adnan Menderes University Faculty of Medicine (protocol code 2026/178; date of approval: 30 April 2026). Informed consent was waived given the retrospective design. All data were de-identified prior to analysis. During the preparation of this manuscript, the author(s) used Claude Sonnet 4.6 (Anthropic, San Francisco, CA, USA) for the purposes of language editing and manuscript drafting assistance. The authors have reviewed and edited the output and take full responsibility for the content of this publication.

## 3. Results

### 3.1. Patient Characteristics

A total of 92 patients were included. Mean age at diagnosis was 66.3 ± 8.8 years; 55 patients (59.8%) were aged ≥65 years. Baseline PSA was available for 72 patients (78.3%), with a median of 78.7 ng/mL (IQR: 36.6–297.1). ISUP grade group 4–5 (Gleason score ≥ 8) was present in 81.5%, and de novo metastatic disease in 81.5%. High-volume metastatic disease by ChemoHormonal Therapy versus Androgen Ablation Randomized Trial for Extensive Disease (CHAARTED) criteria was identified in 45.7%, and high Abiraterone Acetate Plus Prednisone in Patients With Newly Diagnosed High-Risk Metastatic Castration-Naive Prostate Cancer (LATITUDE) risk in 45.7%. Fifty percent received ADT without an ARPI; 39.1% received docetaxel-based chemotherapy. Detailed characteristics are presented in Table 1.

CRPC developed in 66 patients (71.7%); 26 (28.3%) were censored without CRPC. Median time to CRPC was 21.1 months (IQR: 9.3–23.8). Median OS from ADT initiation was 36.3 months; median OS from diagnosis was 37.0 months.

### 3.2. NGS Panel Findings

The most frequently altered genes were ataxia telangiectasia and Rad3-related protein (*ATR*) (35.9%), *PTEN* (28.3%), *TP53* (26.1%), and *BRCA2* (15.2%). *KMT2C* and *MRE11* alterations were detected in 5.4% and 8.7%, respectively. *CDK12* alterations were absent. *RB1*, *ATM*, *SPOP*, and concurrent *TP53*/*RB1* co-alteration (all *n* < 5) were excluded from inferential analyses. At the pathway level, *HRR* deficiency was identified in 53.3%, attributable largely to the high *ATR* frequency. *DDR* deficiency was present in 54.3%, *PI3K/AKT/PTEN* in 43.5%, *AR* signaling in 30.4%, and *MMR* deficiency in 16.3%. *TGF-β* pathway alterations (3.3%) were excluded from survival analyses. The high-risk genomic group was present in 32.6%; 55.4% had high genomic burden (≥2 dysregulated pathways). Findings are summarized in Table 2.

### 3.3. Comparison of Patients with and Without CRPC Development

De novo metastatic disease (77.3% vs. 47.1%, *p* = 0.018), bone metastasis (83.1% vs. 48.5%, *p* = 0.002), high-volume disease by CHAARTED (*p* = 0.019), and high LATITUDE risk (*p* = 0.036) were significantly more frequent in patients who developed CRPC. Age, baseline PSA, ISUP grade, visceral metastasis, and treatment regimen did not differ significantly. None of the genomic variables demonstrated a statistically significant difference between groups in chi-square analyses. Results are presented in Table 3.

### 3.4. Time to CRPC: Kaplan-Meier Analyses

Among clinical variables, de novo metastatic disease (17.9 vs. 65.6 months; *p* = 0.008), high LATITUDE risk (16.3 vs. 25.5 months; *p* = 0.002), high-volume disease versus no metastasis (16.3 vs. 38.8 months; *p* = 0.001), and high ISUP grade (19.2 vs. 73.1 months; *p* = 0.017) were significantly associated with shorter time to CRPC. Younger age (<65 years: 16.8 vs. 24.7 months; *p* = 0.072), baseline PSA ≥ 20 ng/mL (17.9 vs. 41.0 months; *p* = 0.079), and first-line ARPI use (18.5 vs. 24.7 months; *p* = 0.074) showed borderline associations (Table 4).

At the individual gene level, *KMT2C* alteration was associated with significantly shorter time to CRPC (6.4 vs. 21.3 months; *p* = 0.023); variant-level details of all *KMT2C* alterations identified in the cohort are provided in Supplementary Table S1. *TP53* alteration was also associated with shorter time to CRPC (15.5 vs. 23.7 months; *p* = 0.043). *MRE11* alteration demonstrated a borderline association (10.1 vs. 22.7 months; *p* = 0.085). No significant associations were observed for *HRR* deficiency, *DDR* deficiency, *AR* signaling pathway, *ATR*, *CHEK2*, *BRCA2*, *PTEN*, or remaining pathway variables. (Table 4).

**Figure 1:**
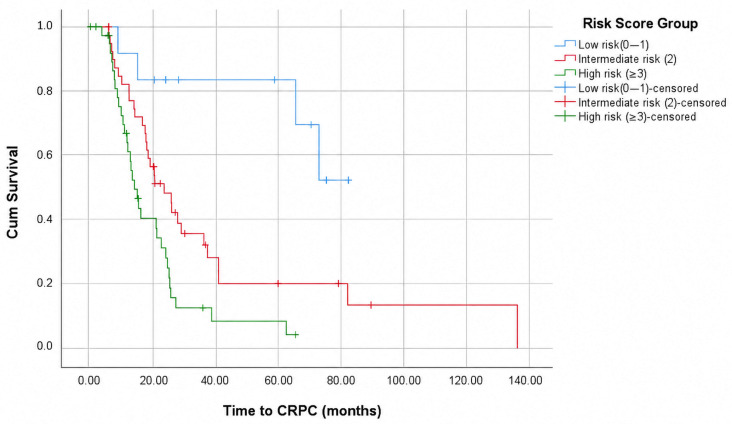
Kaplan-Meier curves for time to CRPC according to the exploratory composite clinical-genomic risk score. Low risk (*n* = 12), Intermediate risk (*n* = 40), High risk (*n* = 40). Overall log-rank *p* < 0.001. Tick marks represent censored observations.

**Figure 2:**
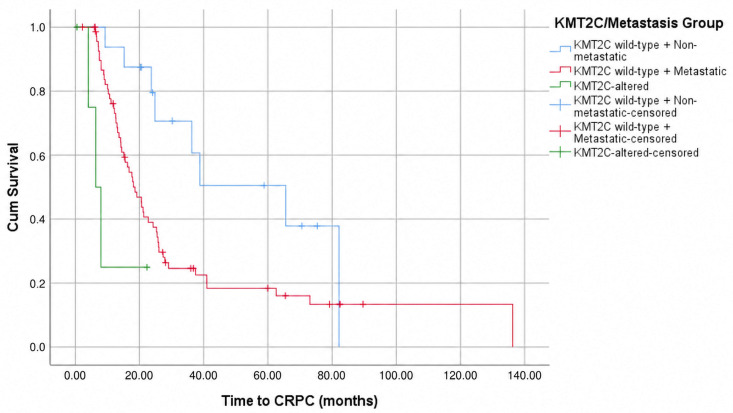
Kaplan-Meier curves for time to CRPC according to *KMT2C* alteration status and metastatic disease. *KMT2C* wild-type non-metastatic (*n* = 16), *KMT2C* wild-type metastatic (*n* = 71), *KMT2C*-altered (*n* = 5). Median: 65.6, 18.5, 6.4 months. Overall log-rank *p* = 0.001. Tick marks represent censored observations.

**Figure 3:**
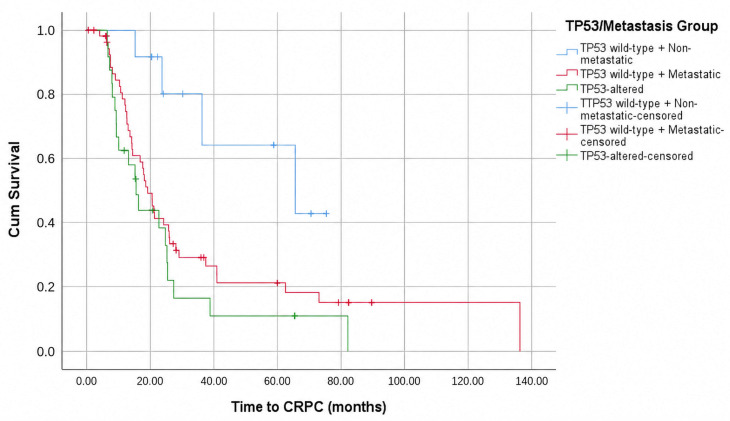
Kaplan-Meier curves for time to CRPC according to *TP53* alteration status and metastatic disease. *TP53* wild-type non-metastatic (*n* = 12), *TP53* wild-type metastatic (*n* = 56), *TP53*-altered (*n* = 24). Median: 65.6, 19.2, 15.5 months. Overall log-rank *p* = 0.003. Tick marks represent censored observations.

Among composite variables, high genomic burden (17.9 vs. 25.9 months; *p* = 0.011) and the high-risk genomic group (15.5 vs. 23.7 months; *p* = 0.031) were significantly associated with shorter time to CRPC (Table 4). The exploratory risk score stratified patients into low (median not reached), intermediate (23.7 months), and high-risk (14.3 months) groups (overall *p* < 0.001), with all pairwise comparisons significant (Figure 1).The *KMT2C*/metastasis group demonstrated a significant stepwise pattern: non-metastatic *KMT2C* wild-type (65.6 months), metastatic *KMT2C* wild-type (18.5 months), and *KMT2C*-altered (6.4 months; overall *p* = 0.001), with significant pairwise differences between the non-metastatic group and both other groups (*p* = 0.015 and *p* = 0.001) (Figure 2). The *TP53*/metastasis group showed a similar pattern (65.6 vs. 19.2 vs. 15.5 months; overall *p* = 0.003) (Figure 3). All results are summarized in Table 4.

### 3.5. Time to CRPC: Cox Regression Analyses

#### 3.5.1. Univariable Analyses

LATITUDE high-risk classification (HR = 2.198, *p* = 0.002), high-volume disease (HR = 3.063, *p* = 0.001), de novo metastatic disease (HR = 2.642, *p* = 0.010), high ISUP grade (HR = 2.338, *p* = 0.020), *KMT2C* alteration (HR = 3.589, *p* = 0.033), *TP53* alteration (HR = 1.720, *p* = 0.045), high genomic burden (HR = 1.916, *p* = 0.012), high-risk genomic group (HR = 1.745, *p* = 0.034), *KMT2C*-altered vs. non-metastatic (HR = 7.446, *p* = 0.003), *TP53*-altered vs. non-metastatic (HR = 4.774, *p* = 0.004), and risk score high vs. low (HR = 7.504, *p* < 0.001) were significantly associated with time to CRPC. *AR* signaling pathway alteration was not significantly associated with time to CRPC (HR = 1.324, 95% CI: 0.762–2.302, *p* = 0.319) (Supplementary Figure S1). Full results are in Table 5.

#### 3.5.2. Multivariable Analyses

Five multivariable models were constructed. Model 2 (metastatic status, ISUP grade, *KMT2C*, *TP53*, genomic burden) demonstrated the best fit (−2LL = 464.300), with de novo metastatic disease (HR = 3.055, *p* = 0.006), *KMT2C* alteration (HR = 6.804, *p* = 0.003), and high genomic burden (HR = 1.917, *p* = 0.032) as independent predictors. Model 4, the most parsimonious, identified *KMT2C* (HR = 6.726, *p* = 0.003) and ISUP grade 4–5 (HR = 2.844, *p* = 0.007) as sole independent predictors. Model 3 confirmed the independent prognostic value of the exploratory risk score (high vs. low: HR = 7.904, *p* = 0.001). Results are summarized in Table 6.

### 3.6. Overall Survival Analyses

#### 3.6.1. Overall Survival from ADT Initiation (OS-ADT)

**Figure 4:**
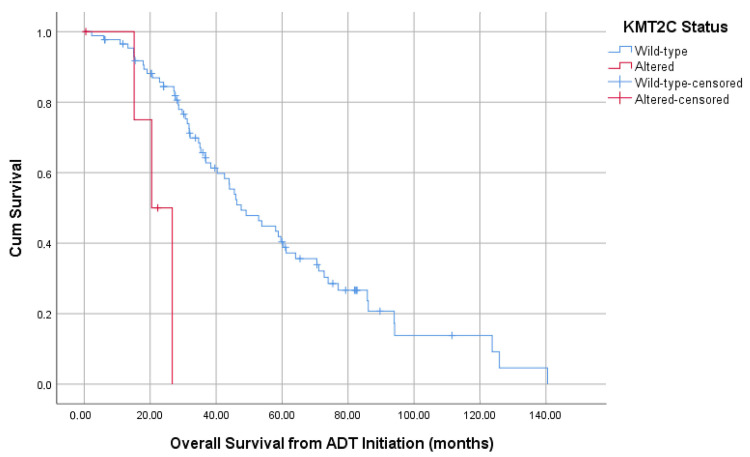
Kaplan-Meier curves for OS from ADT initiation according to *KMT2C* alteration status. *KMT2C* wild-type (*n* = 87) vs. *KMT2C*-altered (*n* = 5). Median OS: 38.4 vs. 20.5 months. Log-rank *p* = 0.005. Tick marks represent censored observations.

*KMT2C* alteration (20.5 vs. 38.4 months; *p* = 0.005) (Figure 4), ISUP grade 4–5 (35.3 vs. 88.5 months; *p* = 0.021), LATITUDE high risk (35.1 vs. 44.0 months; *p* = 0.009), *TP53* alteration (34.7 vs. 44.0 months; *p* = 0.049), the high-risk genomic group (*p* = 0.043), and the risk score (*p* = 0.006) were significantly associated with shorter OS-ADT. *MMR* deficiency showed a borderline association (20.7 vs. 44.0 months; *p* = 0.060). *AR* signaling pathway alteration was not significantly associated with OS-ADT (median 52.9 vs. 43.9 months; HR = 1.491, *p* = 0.186) (Supplementary Figure S2). In multivariable analysis, *KMT2C* (HR = 4.730, *p* = 0.019), *TP53* (HR = 1.810, *p* = 0.038), and ISUP grade 4–5 (HR = 2.364, *p* = 0.050) were independently associated with shorter OS-ADT.

#### 3.6.2. Overall Survival from Diagnosis (OS-Diagnosis)

**Figure 5:**
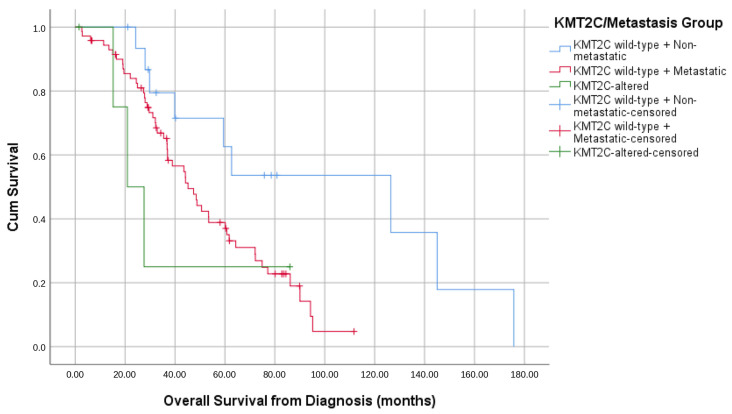
Kaplan-Meier curves for OS from diagnosis according to *KMT2C* alteration status and metastatic disease. *KMT2C* wild-type non-metastatic (*n* = 16), *KMT2C* wild-type metastatic (*n* = 71), *KMT2C*-altered (*n* = 5). Median OS: 126.0, 45.0, 24.0 months. Overall log-rank *p* = 0.048. Tick marks represent censored observations.

ISUP grade 4–5 (43.0 vs. 94.0 months; *p* = 0.002), de novo metastatic disease (44.0 vs. 126.0 months; *p* = 0.006), the *KMT2C*/metastasis group (*p* = 0.044) (Figure 5), and the risk score (*p* = 0.008) were significantly associated with shorter OS-diagnosis. *KMT2C* alteration, *TP53*, and the high-risk genomic group did not reach significance in OS-diagnosis analyses, suggesting their prognostic impact is predominantly confined to the post-ADT phase. *AR* signaling pathway alteration was not significantly associated with OS-diagnosis (median 50.7 vs. 44.2 months; HR = 1.268, *p* = 0.425) (Supplementary Figure S3). In multivariable analysis, ISUP grade 4–5 (HR = 2.504, *p* = 0.029) and *KMT2C*-altered vs. non-metastatic (HR = 5.101, *p* = 0.030) were independent predictors. Results are in Table 7.

## 4. Discussion

This study evaluated whether NGS panel findings obtained during the hormone-sensitive phase of prostate cancer were associated with time to CRPC and overall survival. In our real-world cohort of 92 patients, alterations in *KMT2C* and *TP53*, higher genomic burden, and metastatic disease at diagnosis were independently associated with earlier castration resistance. In contrast, classical *HRR* deficiency was not significantly associated with this endpoint. These results suggest that genomic findings from the hormone-sensitive stage may provide prognostic information beyond routine clinical factors, and could help guide treatment intensification before castration resistance develops.

### 4.1. KMT2C: An Epigenetic Predictor of Early Castration Resistance

*KMT2C* alteration was the strongest genomic predictor in this study. Patients with *KMT2C* alterations developed CRPC at a median of 6.4 months from ADT initiation, compared to 21.3 months in wild-type patients, and this remained independent of metastatic status and ISUP grade in multivariable analysis (HR = 6.804, *p* = 0.003). *KMT2C* was also the strongest independent predictor of OS from ADT initiation (HR = 4.730, *p* = 0.019), with a median OS of 20.5 versus 38.4 months.

These findings are supported by Zhu et al., who reported significantly shorter castration resistance-free survival (9.9 vs. 22.0 months, *p* = 0.015) and OS (71.9 vs. 137.4 months, *p* = 0.012) in *KMT2C*-mutated patients, with *KMT2C* confirmed as an independent predictor of OS (HR = 3.815) [16]. The agreement between two independent cohorts using different biopsy approaches—liquid biopsy versus tissue-based NGS—and from geographically distinct populations strengthens the clinical relevance of this finding. Our cohort showed an even shorter median time to CRPC, which may reflect differences in disease stage at NGS analysis, the earlier disease phase studied, or population-specific characteristics.

The mechanistic basis for this aggressive behavior has been clarified by Guo et al. [17]. Under ADT, *KMT2C* is normally recruited to enhancers of AR-regulated genes to maintain the luminal adenocarcinoma lineage. When *KMT2C* is lost, ASPP2 expression decreases, triggering ΔNp63-dependent transdifferentiation into double-negative prostate cancer (DNPC)—a phenotype lacking both AR and neuroendocrine markers. This DNPC state sustains tumor growth through fatty acid synthesis, bypassing androgen dependence entirely. This mechanism directly explains the rapid CRPC development seen in *KMT2C*-altered patients in our cohort.

To our knowledge, this is the first study to report the prognostic significance of tissue-based *KMT2C* alteration during the hormone-sensitive phase in a Turkish patient cohort, contributing real-world evidence from a population underrepresented in the genomic literature. *KMT2C* testing at the time of initial NGS may identify patients at high risk for early castration resistance who could benefit from treatment intensification or earlier inclusion in biomarker-driven trials.

### 4.2. The HRR Paradox: Why Did HRR Deficiency Fail to Predict CRPC?

One of the unexpected findings of this study was the lack of prognostic significance for *HRR* deficiency, despite its central role in the genomic landscape of advanced prostate cancer. Neither the restricted *HRR* definition nor the broader *DDR* deficiency variable was associated with time to CRPC or overall survival in any analysis.

A key explanation lies in the unusually high *ATR* alteration frequency in our cohort (35.9%), which drove *HRR* deficiency prevalence to 53.3%—well above the 19–28% reported in landmark mCRPC studies [10,13]. *ATR* is a DNA damage-sensing kinase that operates upstream of classical *HRR* effectors; while it is technically grouped within broader *DDR* classifications, its biological role differs substantially from core *HRR* genes such as *BRCA1*/2 and *ATM*. In the PROfound trial, *ATR*-altered patients were placed in Cohort B alongside 12 other non-BRCA/*ATM* genes, where olaparib showed markedly attenuated benefit compared to Cohort A [13]. The enrichment of *ATR* alterations in our cohort therefore introduced substantial heterogeneity into the *HRR* group, likely diluting any true prognostic signal. Consistent with this interpretation, separate analysis of *HRR*_core—comprising only *BRCA1*, *BRCA2*, and *ATM*—also failed to reach significance, though the small number of patients in this subgroup limits definitive conclusions.

Beyond the compositional issue, there is a broader biological argument. *HRR* deficiency may confer therapeutic vulnerability—particularly to PARP inhibitors—without necessarily accelerating the pace of castration resistance during the hormone-sensitive phase. Annala et al. showed that *BRCA2* and *ATM* defects predict resistance to first-line abiraterone and enzalutamide in mCRPC [11], but the castration-sensitive phase represents a biologically distinct context where *HRR* status may carry less prognostic weight. Real-world data support this: Hommerding et al. reported *HRR* mutation prevalence of 20.8% in a mixed primary and mCRPC cohort, with *BRCA2* frequency lower than in landmark mCRPC series, reflecting the stage-dependent enrichment of these alterations [18].

The unexpected direction of *BRCA2* in our Kaplan-Meier analysis—where *BRCA2*-altered patients showed numerically longer time to CRPC—may also reflect the inability to distinguish germline from somatic alterations in our tissue-based panel. Germline *BRCA2* carriers may exhibit a distinct clinical trajectory compared to those with somatic *BRCA2* loss, and the absence of this distinction represents a recognized limitation of retrospective tissue NGS studies.

Taken together, the failure of *HRR* deficiency to predict time to CRPC in our cohort does not contradict its established role in advanced disease, but rather highlights that its prognostic relevance may be phase-dependent and that panel composition—particularly *ATR* inclusion—substantially influences *HRR* prevalence estimates across studies.

### 4.3. TP53 and Genomic Burden: The Case for Cumulative Genomic Damage

Beyond *KMT2C*, both *TP53* alteration and high genomic burden independently predicted a shorter time to CRPC in the multivariable analysis. This finding suggests that castration resistance may reflect the combined effect of alterations across several pathways, rather than the impact of a single gene alone.

*TP53* alteration was present in 26.1% of patients and was associated with shorter time to CRPC (15.5 vs. 23.7 months, *p* = 0.043) and shorter OS from ADT initiation (34.7 vs. 44.0 months, *p* = 0.049). In multivariable analysis incorporating metastatic status, ISUP grade, *KMT2C*, and genomic burden, *TP53* lost independent significance for time to CRPC—suggesting that its prognostic effect is partly shared with other variables in the model. However, it retained independent significance for OS-ADT (HR = 1.810, *p* = 0.038), indicating that *TP53* contributes prognostic information beyond CRPC development alone. This is consistent with findings from Annala et al., who demonstrated that *TP53* defects independently predict primary resistance to abiraterone and enzalutamide in treatment-naive mCRPC [11], and with the mechanistic work of Mu et al. showing that compound *TP53* and *RB1* loss drives lineage plasticity through SOX2 upregulation [12]. Although concurrent *TP53* and *RB1* loss was rare in our cohort (*n* = 2), the independent prognostic impact of *TP53* alone suggests that even partial disruption of p53-mediated tumor suppression is clinically meaningful during the hormone-sensitive phase.

High genomic burden—defined as alterations in two or more dysregulated pathways—was an independent predictor of shorter time to CRPC in our best-fitting multivariable model (HR = 1.917, *p* = 0.032). This finding aligns with the broader principle that genomic complexity, rather than any single alteration, reflects the biological aggressiveness of the disease. Patients with high genomic burden had a median time to CRPC of 17.9 months versus 25.9 months in the low burden group, a difference that remained significant after adjusting for metastatic status, ISUP grade, and *KMT2C*.

The exploratory composite risk score, which combined*KMT2C* mutation, *TP53* mutation, de novo metastatic disease, high ISUP grade, and high genomic burden, provided the clearest separation of risk in this analysis. Patients in the high-risk group had a median time to CRPC of 14.3 months, whereas the median was not reached in the low-risk group (HR = 7.904, *p* = 0.001). Because this score was developed post hoc, it should be tested prospectively before it is used in clinical practice. Still, these findings suggest that combining genomic and clinical features may offer a practical way to stratify patients at diagnosis.

### 4.4. MMR Deficiency: A Temporal Dissociation with Clinical Implications

*MMR* deficiency was identified in 16.3% of patients in this cohort—a rate higher than the 2–5% typically reported in mCRPC series, likely reflecting differences in panel composition, disease phase, and patient selection. Notably, *MMR* deficiency showed a strikingly different pattern from other genomic variables: it did not predict time to CRPC (*p* = 0.843) but was associated with shorter OS from ADT initiation, with a borderline significant difference in median OS of 20.7 versus 44.0 months (*p* = 0.060).

This temporal dissociation—where *MMR* deficiency influences survival but not the pace of castration resistance—is biologically plausible. *MMR*-deficient tumors accumulate somatic mutations at a higher rate, generating a hypermutated phenotype that may not inherently accelerate androgen escape but does progressively reshape the tumor microenvironment and immune landscape over time. The clinical effect of hypermutation may therefore become clearer after CRPC develops, when tumor heterogeneity is greater and immune escape mechanisms are more established.

The practical implication of this finding is significant. Boiarsky et al. demonstrated that *MMR*-deficient mCRPC patients treated with pembrolizumab experienced dramatically improved outcomes compared to *MMR*-proficient patients, with a median radiographic progression-free survival of 54 versus 3.7 months and a median OS not reached versus 17 months [19]. Four patients achieved durable remissions exceeding 2.5 years. These results, combined with the AUA/SUO 2026 guideline recommendation to offer pembrolizumab in dMMR/MSI-H mCRPC [20], establish a clear treatment pathway for this subset.

If *MMR* deficiency does not shorten time to CRPC, these patients may look similar to *MMR*-proficient patients during the hormone-sensitive phase. However, their prognosis after CRPC appears worse, and they may be candidates for immunotherapy once progression occurs. Identifying *MMR*-deficient patients before CRPC could therefore help clinicians plan ahead, including the early assessment of pembrolizumab eligibility at progression and consideration of clinical trials testing immunotherapy combinations with ADT in the hormone-sensitive setting.

### 4.5. AR Signaling Pathway in the Context of Co-Occurring Genomic Alterations

*AR* signaling pathway alterations were found in 30.4% of patients in our cohort, which is in line with previously reported rates in advanced prostate cancer [10]. In our analyses, AR pathway alterations were not independently associated with time to CRPC or overall survival. Still, their presence may be relevant when they occur together with other genomic changes. For example, when AR pathway alterations coexist with *TP53* loss, SOX2-mediated transcriptional changes may support androgen-independent growth and promote lineage plasticity [12]. In *MMR*-deficient tumors, concurrent AR pathway alterations may also influence the tumor microenvironment and could be relevant to immunotherapy response, including pembrolizumab benefit [19,20]. In *HRR*-deficient tumors, AR amplification and splice variant expression have been described as possible mechanisms of resistance to PARP inhibitors [13]. For this reason, AR pathway status may be more informative when assessed together with other alterations rather than on its own. We did not perform formal subgroup analyses according to AR status within the *TP53*, *HRR*, or *MMR*-deficient subgroups because the sample size was too small for reliable comparisons. These possible interactions should be examined in larger prospective studies.

### 4.6. Limitations and Future Perspectives

This study has several limitations. The retrospective single-center design and small sample size limit statistical power, particularly for genomic subgroups with few positive cases such as *KMT2C* (*n* = 5). The inability to distinguish germline from somatic alterations may partly explain unexpected findings such as the non-significant direction of *BRCA2*, and paired germline testing would strengthen future analyses. Treatment heterogeneity across the cohort—including variation in ARPI use and chemotherapy—may also act as a confounding factor despite multivariable adjustment. The composite risk score and high-risk genomic group were constructed posthoc and require independent validation before clinical application.External validation of the present findings, particularly the prognostic role of *KMT2C* alteration, in larger publicly available cohorts such as the TCGA prostate adenocarcinoma dataset or SU2C-funded prostate cancer cohorts represents an important priority for future research.

Despite these limitations, the findings support several priorities for future research. The link between *KMT2C* loss and DNPC transdifferentiation [17] suggests that fatty acid synthesis inhibition may be a rational therapeutic target in *KMT2C*-altered patients. For *MMR*-deficient patients, whether earlier pembrolizumab deployment—in the hormone-sensitive phase—can prevent the poor post-CRPC outcomes observed here warrants prospective evaluation. More broadly, our data support routine NGS testing at ADT initiation not only for PARP inhibitor and immunotherapy eligibility in established mCRPC, but as a tool for proactive risk stratification before castration resistance develops.

## 5. Conclusions

This study demonstrates that NGS panel analysis during the hormone-sensitive phase of prostate cancer provides clinically meaningful prognostic information beyond established clinical parameters. *KMT2C* alteration emerged as the strongest independent genomic predictor of both early castration resistance and overall survival, a finding now supported by mechanistic evidence and corroborated by an independent cohort. *TP53* alteration and high genomic burden were additional independent predictors, highlighting the prognostic value of cumulative genomic dysregulation. Conversely, *HRR* deficiency did not predict time to CRPC, likely due to the compositional influence of high *ATR* alteration frequency in this cohort. *MMR* deficiency demonstrated a clinically important temporal dissociation—not accelerating CRPC development but significantly shortening post-ADT survival—underscoring the value of early genomic identification for timely pembrolizumab planning. Taken together, these findings support the routine integration of NGS testing at ADT initiation as both a prognostic tool and a platform for biomarker-guided treatment decisions in prostate cancer.

## Figures and Tables

**Table 1:** Baseline Patient Characteristics (*n* = 92).

Variable	*n* (%) or Value
Age at diagnosis, years— Mean ± SD/Median (IQR)	66.3 ± 8.8/66.0 (59.0–74.0)
<65/≥65 years	37 (40.2%)/55 (59.8%)
Baseline PSA, ng/mL—Median (IQR) †	78.7 (36.6–297.1)
ISUP Grade Group 1–3/4–5	17 (18.5%)/75 (81.5%)
Metastatic status— Non-metastatic/De novo	17 (18.5%)/75 (81.5%)
Metastatic volume (CHAARTED)— None/Low/High	22 (23.9%)/28 (30.4%)/42 (45.7%)
LATITUDE risk—Low/High	50 (54.3%)/42 (45.7%)
Bone/LN/Visceral metastasis	59 (64.1%)/41 (44.6%)/10 (10.9%)
First-line ARPI— None/Enzalutamide/Abiraterone/Other	46 (50.0%)/24 (26.1%)/19 (20.7%)/3 (3.3%)
First-line chemotherapy— None/Docetaxel/Cisplatin-etoposide	55 (59.8%)/36 (39.1%)/1 (1.1%)
CRPC development—Yes/No (censored)	66 (71.7%)/26 (28.3%)
Time to CRPC, months—Median (IQR) ‡	21.1 (9.3–23.8)
OS from ADT initiation/from diagnosis, months (Median)	36.3/37.0

† Missing for 20 patients (21.7%). ‡ 26 patients censored without CRPC. Abbreviations: SD, standard deviation; IQR, interquartile range; PSA, prostate-specific antigen; ISUP, International Society of Urological Pathology; CHAARTED, ChemoHormonal Therapy versus Androgen Ablation Randomized Trial for Extensive Disease; LATITUDE, Abiraterone Acetate Plus Prednisone in Patients With Newly Diagnosed High-Risk Metastatic Castration-Naive Prostate Cancer; ARPI, androgen receptor pathway inhibitor; LN, lymph node; CRPC, castration-resistant prostate cancer; OS, overall survival; ADT, androgen deprivation therapy.

**Table 2:** NGS Panel Findings (*n* = 92).

Variable	*n* (%)
**Individual Gene Alterations**	
*ATR*/*PTEN*/*TP53*/*BRCA2*	33 (35.9%)/26 (28.3%)/24 (26.1%)/14 (15.2%)
*CHEK2*/*MRE11*/*KMT2C*/*BRCA1* *	9 (9.8%)/8 (8.7%)/5 (5.4%)/5 (5.4%)
*RB1*/*ATM*/*SPOP*/*TP53* + *RB1* concurrent †	4 (4.3%)/3 (3.3%)/2 (2.2%)/2 (2.2%)
*CDK12*	0 (0%)
**Pathway-Level Groups**	
*HRR* deficiency (restricted)/*DDR* deficiency (broad)	49 (53.3%)/50 (54.3%)
*PI3K/AKT*/*PTEN* pathway/*AR* signaling pathway	40 (43.5%)/28 (30.4%)
*MMR* deficiency/*Chromatin modifier*/*WNT* pathway	15 (16.3%)/10 (10.9%)/9 (9.8%)
*TGF-β* pathway †	3 (3.3%)
**Composite Variables**	
High-risk genomic group (*KMT2C*, *TP53*, or *MRE11*)	30 (32.6%)
High genomic burden (≥2 pathways)/Low (0–1)	51 (55.4%)/41 (44.6%)

* Borderline frequency; interpreted with caution. † Excluded from inferential analyses (*n* < 5). Abbreviations: NGS, next-generation sequencing; *HRR*, homologous recombination repair; *DDR*, DNA damage response; PI3K, phosphatidylinositol 3-kinase; AR, androgen receptor; *MMR*, mismatch repair; WNT, wingless-related integration site; TGF-β, transforming growth factor beta; *KMT2C*, lysine methyltransferase 2C.

**Table 3:** Comparison of baseline characteristics between patients with and without CRPC development.

Variable	CRPC No (*n* = 26)	CRPC Yes (*n* = 66)	*p* Value
Age, years (Mean ± SD)	67.0 ± 10.8	66.0 ± 7.9	0.612
Baseline PSA, ng/mL (Median, IQR) †	43.3 (10.2–120.0)	78.7 (36.6–297.1)	0.085
ISUP Grade 4–5	18 (69.2%)	57 (86.4%)	0.075 ‡
De novo metastatic	17 (65.4%)	58 (87.9%)	**0.018 ‡**
High-volume disease (CHAARTED)	7 (26.9%)	35 (53.0%)	**0.019**
Bone metastasis	10 (38.5%)	49 (74.2%)	**0.002**
LATITUDE high risk	7 (26.9%)	35 (53.0%)	**0.036 ‡**
*HRR* deficiency	11 (42.3%)	34 (51.5%)	0.593
*MMR* deficiency	7 (26.9%)	8 (12.1%)	0.116 ‡
*TP53* alteration	4 (15.4%)	20 (30.3%)	0.190 ‡
*KMT2C* alteration	0 (0%)	5 (7.6%)	0.332 ‡
*ATR* alteration	11 (42.3%)	22 (33.3%)	0.473 ‡

† PSA data available for 72 patients only. ‡ Fisher’s Exact Test. Bold values indicate *p* < 0.05. Abbreviations: CRPC, castration-resistant prostate cancer; ISUP, International Society of Urological Pathology; CHAARTED, ChemoHormonal Therapy versus Androgen Ablation Randomized Trial for Extensive Disease; LATITUDE, Abiraterone Acetate Plus Prednisone in Patients With Newly Diagnosed High-Risk Metastatic Castration-Naive Prostate Cancer; *HRR*, homologous recombination repair; *MMR*, mismatch repair.

**Table 4:** Kaplan-Meier Analysis of Time to CRPC (*n* = 92).

Variable	*n*	Median, Months (95% CI)	Log-Rank *p*
**Overall**	92	21.1 (15.4–26.8)	—
**Clinical Variables**			
Metastatic status—Non-met/De novo	17/75	65.6/17.9	**0.008**
LATITUDE—Low/High	50/42	25.5/16.3	**0.002**
CHAARTED—None/Low/High	22/28/42	38.8/18.1/16.3	**<0.001**
ISUP Grade—1–3/4–5	17/75	73.1/19.2	**0.017**
Age <65 vs. ≥65 years	37/55	16.8 vs. 24.7	0.072
PSA ≥ 20 ng/mL (vs <20) †	62/10	17.9 vs. 41.0	0.079
First-line ARPI (Yes vs. No)	46/46	18.5 vs. 24.7	0.074
**Individual Genomic Variables**			
*KMT2C*—WT/Altered	87/5	21.3 (15.6–26.9)/6.4 (2.6–10.2)	**0.023**
*TP53*—WT/Altered	68/24	23.7 (18.3–29.1)/15.5 (11.0–20.0)	**0.043**
*MRE11*—WT/Altered	84/8	22.7 vs. 10.1	0.085
*HRR* deficiency (Neg vs. Pos)	43/49	24.7 (15.6–33.8) vs. 21.1 (10.8–31.3)	0.310
*MMR* deficiency (Neg vs. Pos)	77/15	20.6 (13.6–27.5) vs. 23.7 (8.1–39.1)	0.843
*AR* signaling pathway (Neg vs. Pos)	64/28	21.3 (13.9–28.6)/16.3 (7.6–25.0)	0.317
**Composite Variables**			
Genomic burden—Low/High	41/51	25.9 (14.1–37.7)/17.9 (14.2–21.6)	**0.011**
High-risk genomic group—Neg/Pos	62/30	23.7 (18.3–29.1)/15.5 (10.5–20.5)	**0.031**
*KMT2C*/metastasis: Non-met WT/Met WT/*KMT2C*-alt	16/71/5	65.6/18.5/6.4	**0.001**
*TP53*/metastasis: Non-met WT/Met WT/*TP53*-alt	12/56/24	65.6/19.2/15.5	**0.003**
Risk score—Low/Intermediate/High	12/40/40	NR/23.7 (16.1–31.2)/14.3 (11.0–17.5)	**<0.001**

† PSA data available for 72 patients only. Bold values indicate *p* < 0.05. NR, not reached; WT, wild-type; Non-met, non-metastatic; Met, metastatic; alt, altered.

**Table 5:** Univariable Cox Proportional Hazards Regression for Time to CRPC (*n* = 92).

Variable	HR	95% CI	*p* Value
**Clinical Variables**			
LATITUDE high risk (vs. low)	2.198	1.333–3.623	**0.002**
High-volume disease (vs. no metastasis)	3.063	1.549–6.059	**0.001**
De novo metastatic (vs. non-metastatic)	2.642	1.258–5.550	**0.010**
ISUP Grade 4–5 (vs. 1–3)	2.338	1.143–4.783	**0.020**
Age <65 years (vs. ≥65)	0.637	0.388–1.046	0.075
First-line ARPI (vs. none)	1.561	0.954–2.552	0.076
Baseline PSA ≥ 20 ng/mL (vs. <20) †	2.018	0.907–4.493	0.085
**Genomic Variables**			
*KMT2C* alteration (vs. WT)	3.589	1.107–11.635	**0.033**
*TP53* alteration (vs. WT)	1.720	1.011–2.925	**0.045**
High genomic burden (vs. low)	1.916	1.152–3.186	**0.012**
High-risk genomic group (vs. negative)	1.745	1.044–2.916	**0.034**
*HRR* deficiency (vs. negative)	1.143	0.696–1.877	0.598
*DDR* deficiency (vs. negative)	1.163	0.712–1.901	0.547
*MMR* deficiency (vs. negative)	1.133	0.578–2.220	0.716
*MRE11* alteration (vs. WT)	2.387	0.860–6.624	0.095
*ATR* alteration (vs. WT)	1.425	0.826–2.459	0.203
*CHEK2* alteration (vs. WT)	2.197	0.932–5.180	0.072
*AR* signaling pathway (vs negative)	1.324	0.762–2.302	0.319
**Composite Variables**			
*KMT2C*/met—Metastatic WT (vs. Non-met WT)	2.417	1.147–5.092	**0.020**
*KMT2C*/met—*KMT2C*-altered (vs. Non-met WT)	7.446	1.935–28.650	**0.003**
*TP53*/met—Metastatic WT (vs. Non-met WT)	3.343	1.195–9.348	**0.021**
*TP53*/met—*TP53*-altered (vs. Non-met WT)	4.774	1.624–14.031	**0.004**
Risk score—Intermediate (vs. low)	3.794	1.319–10.909	**0.013**
Risk score—High (vs. low)	7.504	2.570–21.909	**<0.001**

† PSA data available for 72 patients only. Bold values indicate *p* < 0.05. Abbreviations: HR, hazard ratio; CI, confidence interval; WT, wild-type; Non-met, non-metastatic; *HRR*, homologous recombination repair; *DDR*, DNA damage response; *MMR*, mismatch repair; ARPI, androgen receptor pathway inhibitor.

**Table 6:** Multivariable Cox Proportional Hazards Regression for Time to CRPC.

Variable	HR	95% CI	*p* Value
**Model 1—Clinical only (−2LL = 472.636)**			
Age <65 years (vs. ≥65)	0.573	0.344–0.955	**0.033**
LATITUDE high risk (vs. low)	1.740	0.988–3.065	0.055
**Model 2—Clinical + Genomic (−2LL = 464.300)**			
De novo metastatic (vs. non-metastatic)	3.055	1.373–6.795	**0.006**
*KMT2C* alteration (vs. WT)	6.804	1.882–24.601	**0.003**
High genomic burden (vs. low)	1.917	1.056–3.480	**0.032**
ISUP Grade 4–5 (vs. 1–3)	1.986	0.919–4.294	0.081
**Model 3—Risk Score (−2LL = 470.522)**			
Risk score—Intermediate (vs. low)	3.944	1.236–12.586	**0.020**
Risk score—High (vs. low)	7.904	2.260–27.644	**0.001**
**Model 4—Two-variable: *KMT2C* + ISUP (−2LL = 479.261)**			
*KMT2C* alteration (vs. WT)	6.726	1.898–23.840	**0.003**
ISUP Grade 4–5 (vs. 1–3)	2.844	1.335–6.059	**0.007**
**Model 5—High-risk genomic group (−2LL = 474.804)**			
High-risk genomic group (vs. negative)	1.827	1.083–3.085	**0.024**
De novo metastatic (vs. non-metastatic)	2.450	1.135–5.289	**0.022**

Bold values indicate *p* < 0.05. −2LL, −2 log-likelihood (lower = better fit). Abbreviations: HR, hazard ratio; CI, confidence interval; WT, wild-type; ISUP, International Society of Urological Pathology.

**Table 7:** Overall Survival Analyses: Kaplan-Meier and Cox Regression Results.

Variable	Median OS (mo)	95% CI	Log-Rank *p*	HR	*p*
**A. OS-ADT (Kaplan** **-Meier)**					
*KMT2C*—WT/Altered	38.4/20.5	27.6–49.2/18.9–22.1	**0.005**	6.144	**0.005**
ISUP Grade 1–3/4–5	88.5/35.3	—/28.5–42.1	**0.021**	2.871	**0.010**
LATITUDE—Low/High	44.0/35.1	34.8–53.2/27.0–43.2	**0.009**	1.985	**0.009**
*TP53*—WT/Altered	44.0/34.7	29.3–58.7/25.0–44.4	**0.049**	1.719	**0.049**
*MMR* deficiency—Neg/Pos	44.0/20.7	34.8–53.2/20.1–21.3	0.060	1.998	0.060
*AR* signaling pathway—Neg/Pos	52.8 /43.9	35.9–69.8/32.8–55.0	0.183	1.491	0.186
**Multivariable Model A2 (OS-ADT)**					
*KMT2C* alteration	—	1.293–17.304	—	4.730	**0.019**
*TP53* alteration	—	1.035–3.165	—	1.810	**0.038**
ISUP Grade 4–5	—	1.001–5.580	—	2.364	**0.050**
**B. OS-diagnosis (Kaplan-Meier)**					
ISUP Grade 1–3/4–5	94.0/43.0	78.8–109.2/33.8–52.2	**0.002**	3.287	**0.004**
Non-metastatic/De novo metastatic	126.0/44.0	7.9–244.1/34.1–53.9	**0.006**	3.117	**0.009**
*KMT2C*/met: Non-met WT/Met WT/*KMT2C*-alt	126.0/45.0/24.0	41.1—211.8/33.7–56.3/14.3–33.7	**0.048**	4.221/2.726	**0.044/0.022**
*AR* signaling pathway—Neg/Pos	50.6/44.1	34.2–67.1/33.7–54.6	0.424	1.268	0.425
**Multivariable (OS-diagnosis)**					
ISUP Grade 4–5	—	1.101–5.694	—	2.504	**0.029**
*KMT2C*-altered vs. Non-met WT	—	1.172–22.204	—	5.101	**0.030**

Bold values indicate *p* < 0.05. WT, wild-type; Non-met, non-metastatic; Met, metastatic; alt, altered. Abbreviations: OS, overall survival; ADT, androgen deprivation therapy; HR, hazard ratio; CI, confidence interval; ISUP, International Society of Urological Pathology; *TP53*, tumor protein p53; *MMR*, mismatch repair; *KMT2C*, lysine methyltransferase 2C.

## Data Availability

The data presented in this study are available on request from the corresponding author due to privacy and ethical restrictions.

## Supplementary Materials

The following supporting information can be downloaded at https://www.mdpi.com/article/10.3390/curroncol33070416/s1, Figure S1: Kaplan–Meier curves for time to castration-resistant prostate cancer (CRPC) according to *AR* signaling pathway alteration status; Figure S2: Kaplan–Meier curves for overall survival from ADT initiation according to *AR* signaling pathway alteration status; Figure S3: Kaplan–Meier curves for overall survival from prostate cancer diagnosis according to *AR* signaling pathway alteration status.; Table S1: Variant-Level Details of KMT2C Alterations Identified in the Study Cohort (*n*= 5 patients).

## Abbreviations

The following abbreviations are used in this manuscript:

ADT	Androgen deprivation therapy
AR	Androgen receptor
ARPI	Androgen receptor pathway inhibitor
*ATR*	Ataxia telangiectasia and Rad3-related protein
CRPC	Castration-resistant prostate cancer
*DDR*	DNA damage response
DNPC	Double-negative prostate cancer
FFPE	Formalin-fixed paraffin-embedded
*HRR*	Homologous recombination repair
HR	Hazard ratio
IQR	Interquartile range
ISUP	International Society of Urological Pathology
KM	Kaplan-Meier
mCRPC	Metastatic castration-resistant prostate cancer
mHSPC	Metastatic hormone-sensitive prostate cancer
*MMR*	Mismatch repair
NGS	Next-generation sequencing
OS	Overall survival
OS-ADT	Overall survival from ADT initiation
PCa	Prostate cancer
PCWG3	Prostate Cancer Working Group 3
PSA	Prostate-specific antigen
SD	Standard deviation
UMI	Unique molecular identifier
