# Supplementary material for: NGS-Based Genomic Profiling Identifies Independent Predictors of Time to Castration Resistance in Hormone-Sensitive Prostate Cancer: A Retrospective Real-World Study
Source: Curr Oncol. 2026 Jul 10;33(7):416. doi: 10.3390/curroncol33070416 (PMC13408311; doi:10.3390/curroncol33070416)
Supplement: Supplementary file 1 [file curroncol-33-00416-s001.zip › Supplementary Table S1.pdf]

Supplementary Table S1. Variant-Level Details of KMT2C Alterations Identified in the Study Cohort (*n*=5 patients).

**Patient No**	**Gene**	**Exon**	**Nucleotide Change (c.)**	**Protein Change (p.)**	**Mutation Type**	**Classification**
1	*KMT2C*	7	c.925C>T	p.P309S	Missense	Pathogenic
1	*KMT2C*	15	c.2573G>T	p.W858L	Missense	Likely Pathogenic
1	*KMT2C*	18	c.2917A>G	p.R973G	Missense	Likely Pathogenic
2	*KMT2C*	38	c.8390del	p.K2797Rfs*26	Frameshift	Pathogenic
3	*KMT2C*	38	c.8390del	p.K2797Rfs*26	Frameshift	Pathogenic
4	*KMT2C*	38	c.8390del	p.K2797Rfs*26	Frameshift	Pathogenic
5	*KMT2C*	38	c.8390del	p.K2797Rfs*26	Frameshift	Pathogenic

Patient numbers are anonymized. Nucleotide and protein changes are reported according to HGVS nomenclature (NM_170606.3). Variant classification was determined using the QCI-Interpret bioinformatics software (QIAGEN) based on ACMG/AMP criteria.

*Abbreviations: c., coding DNA sequence; p., protein sequence; del, deletion; fs, frameshift; *, stop codon position; P, pathogenic; LP, likely pathogenic.*
