# Supplementary figures and images for: NGS-Based Genomic Profiling Identifies Independent Predictors of Time to Castration Resistance in Hormone-Sensitive Prostate Cancer: A Retrospective Real-World Study
Source: Curr Oncol. 2026 Jul 10;33(7):416. doi: 10.3390/curroncol33070416 (PMC13408311; doi:10.3390/curroncol33070416)

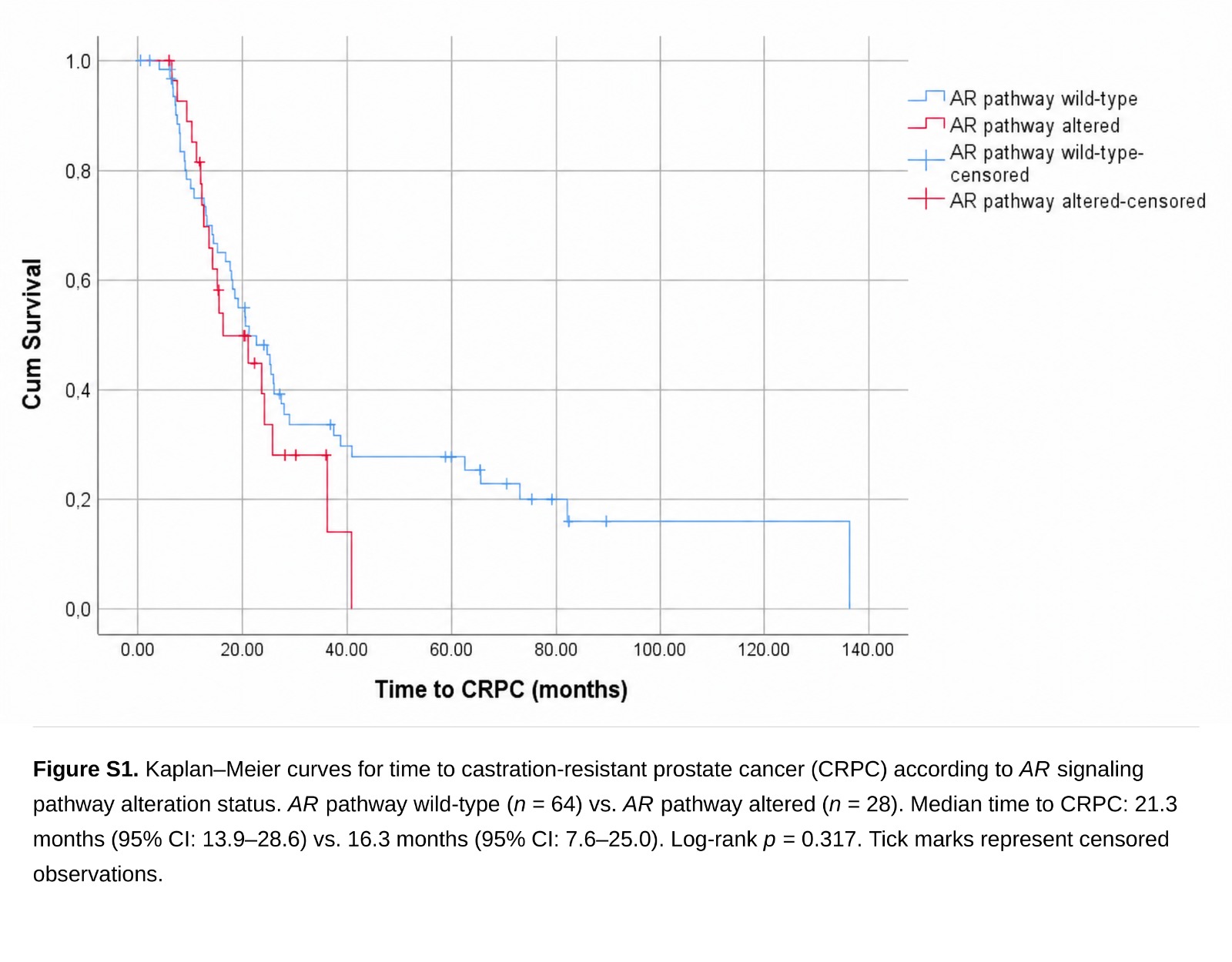

Supplement: Supplementary file 1 [file curroncol-33-00416-s001.zip › Figure S1.jpeg]

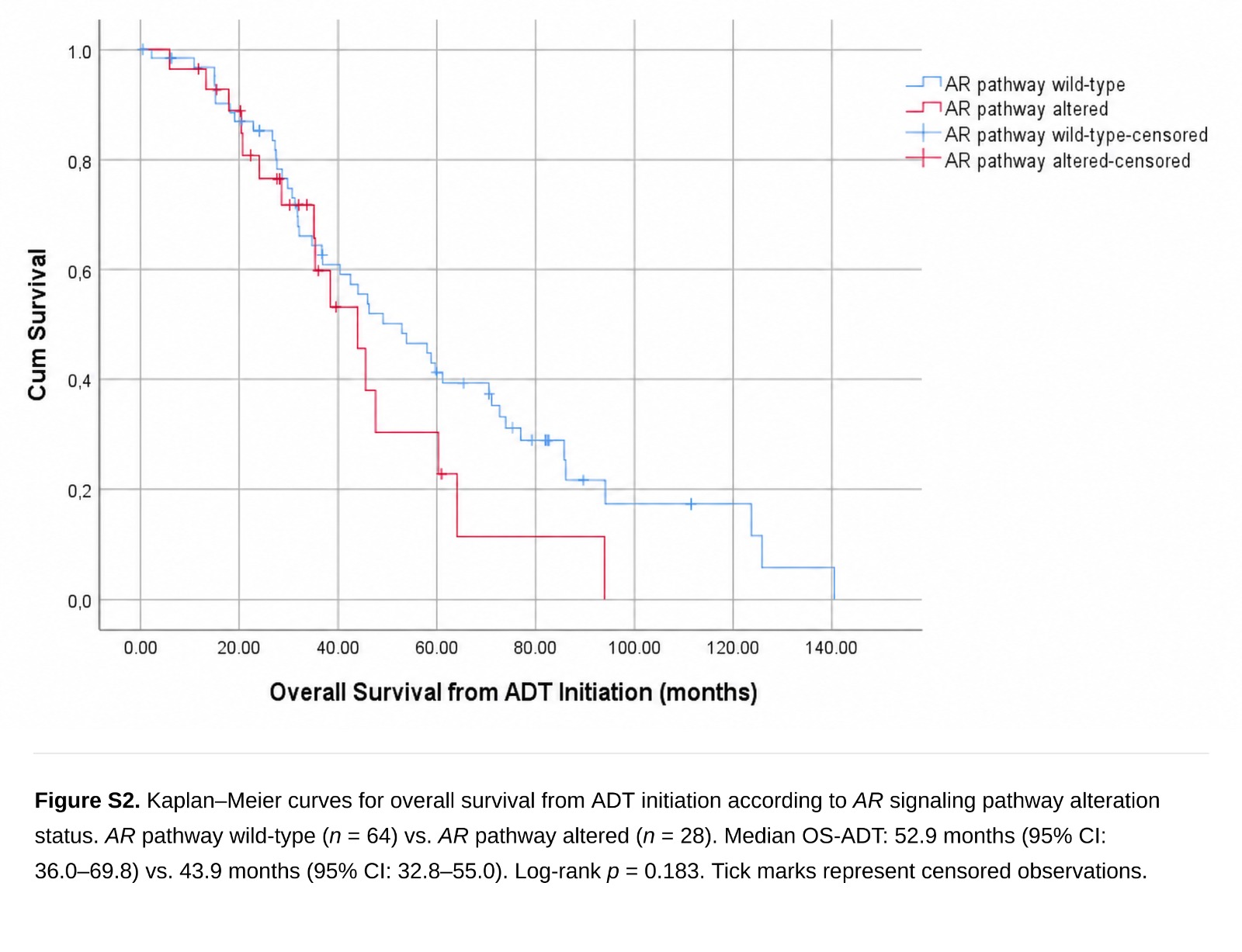

Supplement: Supplementary file 1 [file curroncol-33-00416-s001.zip › Figure S2.jpeg]

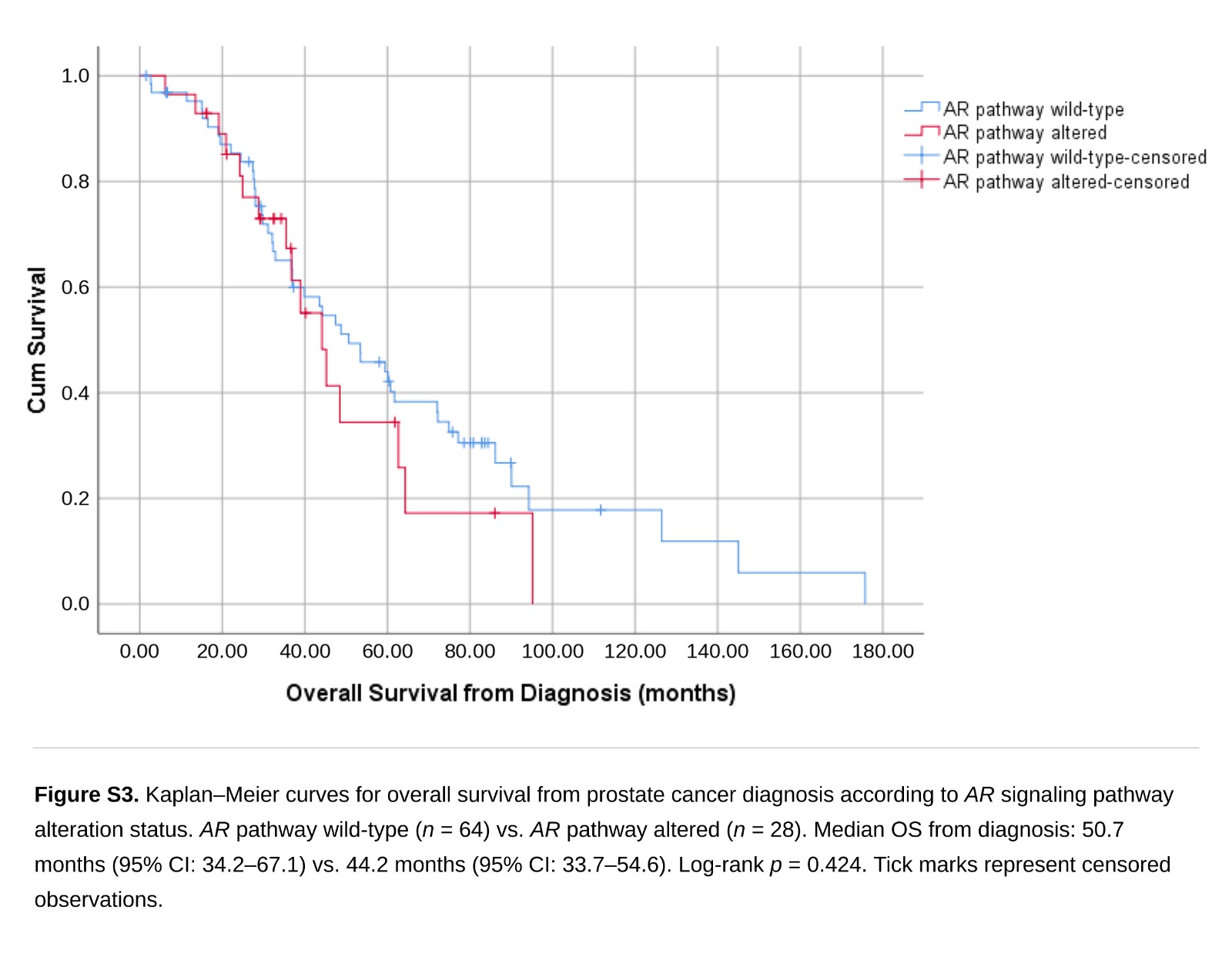

Supplement: Supplementary file 1 [file curroncol-33-00416-s001.zip › Figure S3.jpeg]
